# Supplementary material for: Novel Driver Strength Index highlights important cancer genes in TCGA PanCanAtlas patients
Source: PeerJ. 2022 Aug 11;10:e13860. doi: 10.7717/peerj.13860 (PMC9375969; doi:10.7717/peerj.13860)
Supplement: Supplemental Information 2 [file peerj-10-13860-s002.zip › COHORTS/genes plots/cohorts/2021_8_16_14_9_distribution_cohorts_males_SKCM.pdf]

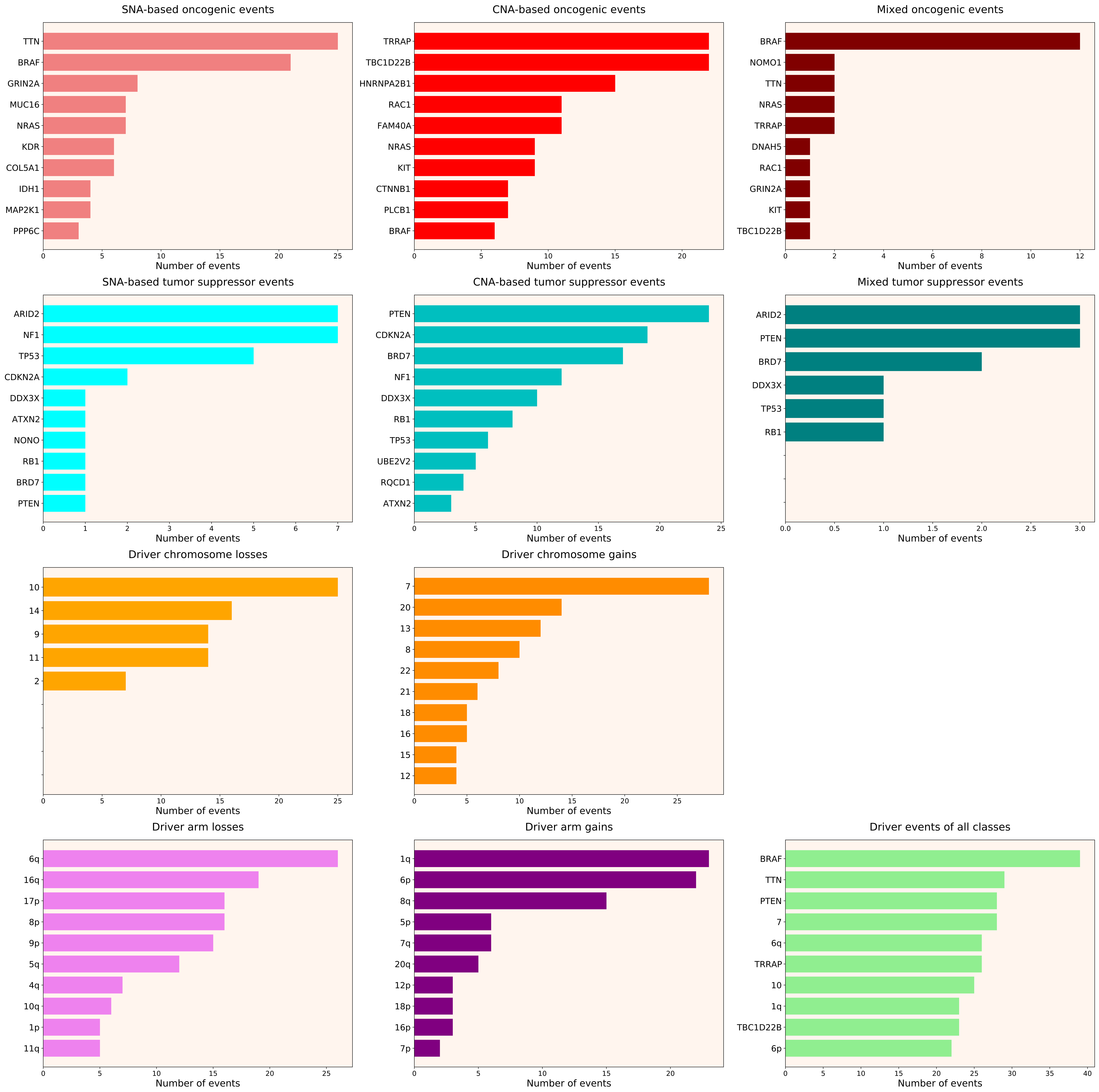
SNA-based oncogenic events
TTN
BRAF
GRIN2A
MUC16
NRAS
KDR
COL5A1
IDH1
MAP2K1
PPP6C
Number of events
CNA-based oncogenic events
TRRAP
TBC1D22B
HNRNPA2B1
RAC1
FAM40A
NRAS
KIT
CTNNB1
PLCB1
BRAF
Number of events
Mixed oncogenic events
BRAF
NOMO1
TTN
NRAS
TRRAP
DNAH5
RAC1
GRIN2A
KIT
TBC1D22B
Number of events
SNA-based tumor suppressor events
ARID2
NF1
TP53
CDKN2A
DDX3X
ATXN2
NONO
RB1
BRD7
PTEN
Number of events
CNA-based tumor suppressor events
PTEN
CDKN2A
BRD7
NF1
DDX3X
RB1
TP53
UBE2V2
RQCD1
ATXN2
Number of events
Mixed tumor suppressor events
ARID2
PTEN
BRD7
DDX3X
TP53
RB1
Number of events
Driver chromosome losses
10
14
9
11
2
Number of events
Driver chromosome gains
7
20
13
8
22
21
18
16
15
12
Number of events
Driver arm losses
6q
16q
17p
8p
9p
5q
4q
10q
1p
11q
Number of events
Driver arm gains
1q
6p
8q
5p
7q
20q
12p
18p
16p
7p
Number of events
Driver events of all classes
BRAF
TTN
PTEN
7
6q
TRRAP
10
1q
TBC1D22B
6p
Number of events
